# Invasive rat eradication strongly impacts plant recruitment on a tropical atoll

**DOI:** 10.1371/journal.pone.0200743

**Published:** 2018-07-17

**Authors:** Coral A. Wolf, Hillary S. Young, Kelly M. Zilliacus, Alexander S. Wegmann, Matthew McKown, Nick D. Holmes, Bernie R. Tershy, Rodolfo Dirzo, Stefan Kropidlowski, Donald A. Croll

**Affiliations:** 1 Ecology and Evolutionary Biology Department, University of California Santa Cruz, Santa Cruz, California, United States of America; 2 Department of Ecology, Evolution and Marine Biology, University of California, Santa Barbara, Santa Barbara, California, United States of America; 3 Botany Department, University of Hawaii at Manoa, Honolulu, Hawaii, United States of America; 4 Island Conservation, Santa Cruz, California, United States of America; 5 Department of Biology, Stanford University, Stanford, California, United States of America; 6 US Fish & Wildlife Service, Honolulu, Hawaii, United States of America; Universita degli Studi di Napoli Federico II, ITALY

## Abstract

Rat eradication has become a common conservation intervention in island ecosystems and its effectiveness in protecting native vertebrates is increasingly well documented. Yet, the impacts of rat eradication on plant communities remain poorly understood. Here we compare native and non-native tree and palm seedling abundance before and after eradication of invasive rats (*Rattus rattus*) from Palmyra Atoll, Line Islands, Central Pacific Ocean. Overall, seedling recruitment increased for five of the six native trees species examined. While pre-eradication monitoring found no seedlings of *Pisonia grandis*, a dominant tree species that is important throughout the Pacific region, post-eradication monitoring documented a notable recruitment event immediately following eradication, with up to 688 individual *P*. *grandis* seedlings per 100m^2^ recorded one month post-eradication. Two other locally rare native trees with no observed recruitment in pre-eradication surveys had recruitment post-rat eradication. However, we also found, by five years post-eradication, a 13-fold increase in recruitment of the naturalized and range-expanding coconut palm *Cocos nucifera*. Our results emphasize the strong effects that a rat eradication can have on tree recruitment with expected long-term effects on canopy composition. Rat eradication released non-native *C*. *nucifera*, likely with long-term implications for community composition, potentially necessitating future management interventions. Eradication, nevertheless, greatly benefitted recruitment of native tree species. If this pattern persists over time, we expect long-term benefits for flora and fauna dependent on these native species.

## Introduction

Non-native rodents have invaded about 80% of the world’s island groups [1], posing a severe threat to native insular biodiversity. Invasive rats (primarily *Rattus rattus*, *R*. *exulans*, *R*. *norvegicus*) are omnivores with a range of direct and indirect impacts on island communities. As predators of animals, they have caused the extinction of numerous animal species in insular communities, including reptiles, seabirds, landbirds and invertebrates [2–4], with many additional indirect impacts, such as effects to nutrient subsidies supplied by these animals [5–7]. In addition, as omnivores, non-native, invasive rats also consume seeds, seedlings, and adult plants [8–12], leading to changes in the abundance, composition, and structure of plant communities [13–15], including the extinction of some endemic island plant species [16].

Invasive rat eradication on islands is an established conservation tool that has been shown to benefit native biodiversity and human well-being [17, 18]. To date, over 650 *Rattus* spp. eradications have been attempted on islands worldwide [19] with many examples of recovery of native animal populations post-eradication [2, 20–26]. Yet, despite the increasing frequency of rat eradications globally and substantial evidence that rats can directly and indirectly affect many plant species, there are few detailed pre- and post-eradication studies within the English scientific literature on plant community change following a rat eradication, especially on tropical islands [27]. Amongst those that have, only a handful of studies–all from temperate New Zealand–have examined the effects of rat removal on seedling recruitment [8, 13, 28–30]. Here we examine the effects of an atoll-wide eradication of black rats (*R*. *rattus*) on the recruitment of native trees and non-native trees and palms on tropical Palmyra Atoll (Northern Line Islands) in the Central Pacific.

Plant species can be expected to vary in their susceptibility to seed predation and herbivory by *Rattus* due to factors such as palatability, seed size (preference for relatively small seeds), accessibility, and seed coat strength [31]. On Palmyra Atoll, invasive *Rattus* species are recognized as seed predators of seven of the eight species of tree and palm included in this study (i.e., *Barringtonia asiatica*, *Calophyllum inophyllum*, *Cocos nucifera*, *Cordia subcordata*, *Guettarda speciosa*, *Hernandia sonora*, *Neisosperma oppositifolium*, *and Pisonia grandis)* [16, 32–34]; the exception is the uncommon, non-native tree, *C*. *inophyllum*, for which rats have been observed manipulating but not killing any seeds [33].

*Rattus rattus* are known to have a particular preference for seeds of *P*. *grandis* [33, 35], an ecologically important native tree that provides nesting habitat for many seabird species [36] as well as important habitat for geckos and insects [37, 38]. Due to a combination of rat and land crab predation, recruitment and establishment of *P*. *grandis* from seed on Palmyra Atoll was very limited (nearly absent) in the presence of rats (H.S. Young unpublished data). Indeed, in an experimental study, Young et al. [39] found that ~99% of all outplanted *P*. *grandis* seeds failed to survive one month. In spite of this low recruitment and survival, the size of the *P*. *grandis* population across Palmyra Atoll has remained one of the largest in the tropical Pacific [33, 40–42], with its persistence likely the result of vegetative sprouting (e.g., plants regenerating through fallen branches). However, a number of investigators have reported that *P*. *grandis* is in decline both on Palmyra Atoll and globally [41–43].

The coconut palm, *C*. *nucifera*, was first spread throughout the Pacific Ocean by Austronesian voyagers over 1500 years ago, then by early European explorers, and most recently by early European agricultural entrepreneurs for copra production [44, 45]. On islands where they have been introduced, coconut palms often form monodominant stands and have been shown to directly compete with *P*. *grandis* for water resources [46]. *Cocos nucifera* was likely introduced to Palmyra Atoll within the past 1500 years [44, 47] with abundance increasing between 1850 and 1970 due to small-scale cultivation efforts [45]. *Rattus rattus* can be an important predator of coconut palm seeds and seedlings (by consuming the sprouting apical meristem); these behaviors may have partially controlled the recent expansion of this invasive plant further into the atoll’s remaining native species forest stands [33, 48]. Thus, while rats are likely directly detrimental to the persistence of native plants on Palmyra Atoll, they may also indirectly benefit native plants by inhibiting the spread of *C*. *nucifera* via consumption of coconuts and germinating plants [49].

In an effort to benefit native plant and animal communities, invasive *R*. *rattus* were eradicated from Palmyra in June 2011 [50], providing the opportunity to investigate plant responses to the rat eradication. Quantifying seedling numbers before and after the rat eradication, we examine the effect of *R*. *rattus* on the establishment of native and non-native plant communities, with the ultimate goal of understanding the effects of rat eradication on tropical plant communities and, in particular, short-term regeneration of both native and non-native plants. Specifically, we: 1) surveyed 55 seedling transects to examine recruitment of common native trees and introduced coconut palms across the atoll, and 2) conducted directed searches of seedlings under adult individuals of rare native and non-native tree species across the atoll.

## Methods

### Study area

**Fig 1:**
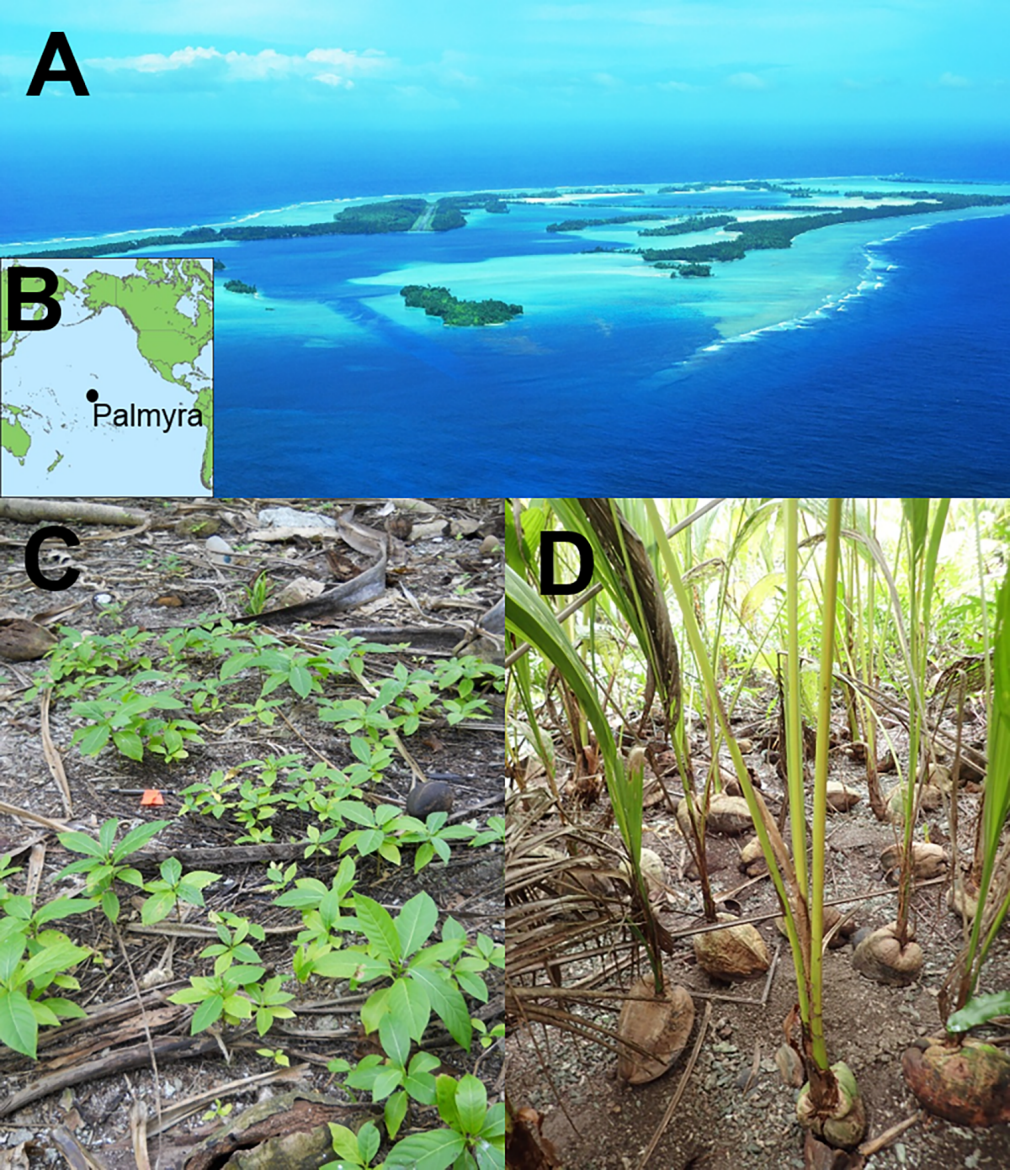
Study site and two plant species that significantly increased post-eradication (*Pisonia grandis* and *Cocos nucifera*). (A) All research was conducted on the islets of Palmyra Atoll, (B) a tropical island located in the Line Islands Chain in the Central Pacific Ocean. (C) The highest density of *P*. *grandis* seedlings was observed one month post- rat eradication, in 2011 and (D) the highest density of non-native *C*. *nucifera* seedlings was observed in 2016, five years post- rat eradication. Reprinted under a CC BY license, with permission from Kydd Pollock (1A), Coral Wolf (1C), and Dena Spatz (1D), original copyright 2009 (1A) and 2016 (1C and 1D).

Palmyra Atoll (5°53′N 162°5′W) is located in the Northern Line Island Chain in the Central Pacific Ocean approximately 1,600 km southwest of the Hawaiian Islands (Fig 1A and 1B) and is co-managed as a U.S. National Wildlife Refuge by the U.S. Fish and Wildlife Service (USFWS) and The Nature Conservancy (TNC). The atoll consists of 25 islets, comprising 235 ha of emergent land dominated by native Pacific tropical island tree species (e.g. *P*. *grandis*) and non-native coconut palms. The site is a wet tropical habitat with limited seasonality and a mean annual temperature and rainfall of 25°C and 4,445 mm, respectively. Non-native *R*. *rattus* were likely introduced to the atoll in the 1940’s during World War II [51].

A closed canopy forest covers much of Palmyra and is comprised of coastal species found across the Pacific. It is dominated by three forest types: (1) monodominant stands of non-native *C*. *nucifera* (“Non-native” forest), (2) mixed species forest of ten native tree and shrub species (dominated by *P*. *grandis*, *Pandanus fischerianus*, and *Heliotropium foertherianum*) and with intermediate densities of non-native *C*. *nucifera* (“Mixed” forest), and (3) native-dominated forest (primarily *P*. *grandis* trees in the interior and *H*. *foertherianum* along the coast), with low abundances of *C*. *nucifera* (“Native” forest) [52].

In 2011, the USFWS and TNC partnered with Island Conservation to eradicate *R*. *rattus* from Palmyra Atoll using an anticoagulant rodenticide, brodifacoum [50]. Rats were functionally extirpated from the atoll (i.e. >99% of the rodent population eliminated) in July 2011, one month after the first rodenticide bait application (June 2011). Rats were confirmed eradicated one year post-eradication (June 2012) following intensive monitoring for the presence of rats across the entire atoll [50]. Brodifacoum is not known to affect mortality of land crabs [53, 54], a prevalent native seed and seedling predator on Palmyra Atoll. In addition, brodifacoum is highly insoluble in water [55], and there is no mechanism to suggest that the anticoagulant impacts plant recruitment or growth.

We conducted pre-eradication monitoring of plant communities in 2004 and 2007 and repeated the monitoring post-eradication in 2011, 2012, 2014, and 2016. We considered the 2011 monitoring, one-month post-eradication, to have measured the immediate impacts of the eradication, and the 2012, 2014, and 2016 monitoring to have measured the initiation of short-term recovery processes. Although there is no regular seasonality in fruiting and seedling germination for the species studied, plant monitoring occurred between June and August in all years. We obtained appropriate permits from USFWS for all work conducted in the refuge (permit numbers: 12533–04003, 12533–07007, 12533–11003, 12533–12003, 12533–14033, and 12533–16016).

### Seedling transects

**Fig 2:**
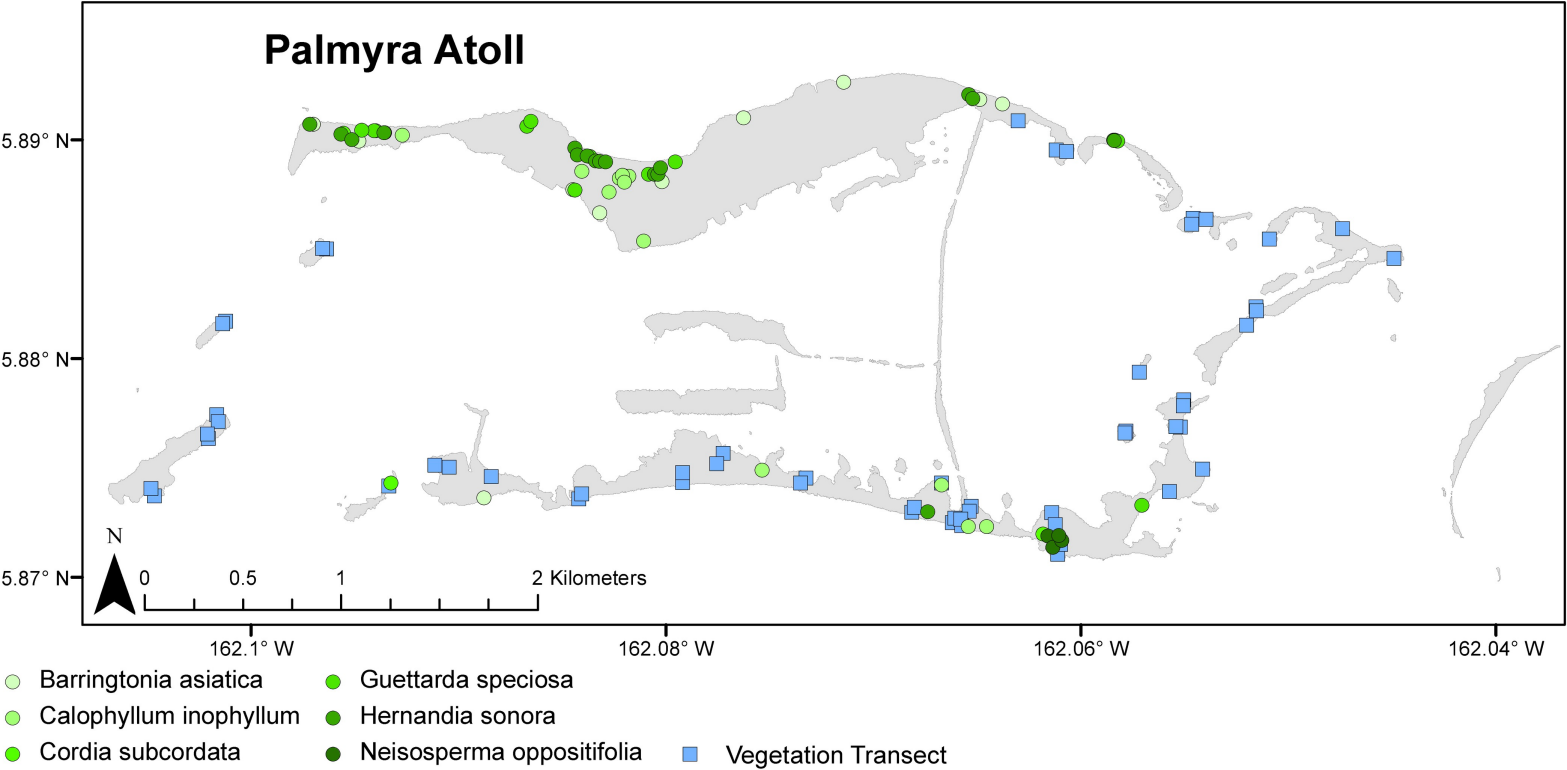
Map of seedling recruitment survey locations on Palmyra Atoll. Map of Palmyra Atoll with locally rare tree seedling plots shown as colored circles and light green squares indicating seedling transects across the atoll.

To examine the effects of *R*. *rattus* eradication on seedling recruitment of common tree species, we conducted repeated surveys of seedling abundance on 55 strip transects distributed across the atoll in 2007 (pre-eradication), and in 2011, 2012, 2014, and 2016 (post-eradication; Fig 2). Strip transects (50 x 2 m) were spaced evenly (by distance along the coast) around 13 of the 25 uninhabited islets [52]. To avoid confounding variables, the one inhabited and largest islet was not sampled. Strip transects were paired and located parallel to the coastline, with one transect (‘coastal’) located 5 m from the high tide line and the second transect (‘inland’) located 50 m inland from the coastal transect. We aimed to place up to 10 transects per islet, while maintaining a minimum of 200 m between coastal transects and not allowing overlap between inland transects. If an islet was less than 100 m wide, the inland transect was located at the centermost point of the island. If an islet was less than 50 m wide, an inland transect was not included. In practice, this minimum distance meant that for small islets there were many with fewer than 10 transects, and the actual number of transects per islet ranged from 1 to 10. Strip transects were re-located each year using a hand-held GPS with an error of up to 5 m. Young et al. (2010) classified transects established prior to the eradication (2007) into one of three forest types based upon the percent basal area of *C*. *nucifera*: “Non-Native”: > 75% basal area of *C*. *nucifera* (n = 24); “Mixed”: ≤ 75% and ≥ 25% basal area of *C*. *nucifera* (n = 14); and “Native” <25% basal area of *C*. *nucifera* (n = 17; details in [52]).

We counted seedlings of both *P*. *grandis* and *C*. *nucifera* rooted within (i.e., at least 50% of the plant’s base) each strip transect. There was no minimum seedling size, and all germinants and cotyledonous seedlings encountered were included in counts. For *P*. *grandis*, we defined “seedlings” arbitrarily as plants less than 40 cm in height, measured from the ground to the apical meristem of the plant. We did not count vegetative sprouting (e.g. plants regenerating from fallen branches) as seedlings. For *C*. *nucifera*, which often exceeded 40 cm before a single leaf opened, we defined “seedlings” as nuts that had germinated but did not have any fully opened fronds. Thus, unlike *P*. *grandis*, *C*. *nucifera* plants ≥ 40 cm in height could still be considered seedlings. All of the *P*. *grandis* seedlings counted on surveys conducted one-month post-eradication (August 2011) were < 10 cm tall, suggesting that these seedlings were most likely from seeds that germinated no more than two months before the survey period (the start of the eradication operation) (E. Adkins unpublished data) [56]. Three native, locally common tree species were excluded from analyses because either they did not generally recruit in the habitats sampled (i.e., coastal species: *Scaevola sericea* and *H*. *foertherianum)* or different counting methods were applied (*P*. *fischerianus*).

### Locally rare tree seedling plots

To assess changes in abundance of less common species, in 2004 (pre-eradication), and in 2011, 2012, 2014, and 2016 (post-eradication), we conducted directed searches for adult individuals (≥ 4m in height) of most known locally rare tree species (defined as approximately < 50 individual adult plants across the atoll; Fig 2). These included five native species (*B*. *asiatica*, *C*. *subcordata*, *H*. *sonora*, *G*. *speciosa*, and *N*. *oppositifolium*) and one non-native tree species, *C*. *inophyllum*. Only one rare native tree species, *Premna serratifolia*, was excluded from surveys because most individuals (five of six) were inaccessible due to their proximity to a sensitive seabird breeding colony. All individual trees observed were mapped and, in 2011, individually identified and marked using permanent aluminum tree tags. For each adult tree located, we established 15 m radius plots around the tree trunk and counted all seedlings (here defined as any plant < 200 cm in total height) of the focal tree species that occurred within the plot. When two or more conspecific rare adult trees occurred within 15 m of one another, we chose the largest trunk within the group as the center point of the radius and created one plot for the group. We surveyed plots around a total of 49, 55, 55, 54, and 53 individual trees in 2004, 2011, 2012, 2014, and 2016 respectively (see S1 Table for species details). Due to adult tree growth and mortality, plot localities and number of plots are not identical across years. In addition, we were unable to track the same tree across the sampling years 2004 and 2011, therefore seedling data were pooled by species by year prior to analysis.

### Analyses

As no *P*. *grandis* seedlings were counted in transects pre-eradication, and post eradication *P*. *grandis* data were not normally distributed, we used a resampling approach. For each year post-eradication, we sampled 10,000 means from the data with replacement, and compared the means to zero (pre-eradication value).

We conducted repeated measures analyses of variance for *C*. *nucifera* seedlings counted in seedling transects using a repeated measure analysis. *Cocos nucifera* data were not normally distributed, so we used log_10_ transformed data to conduct the analyses. To account for sphericity, we used the univariate Greenhouse–Geisser (Univar G-G) correction. To examine individual differences across sampling years, we conducted a series of repeated measures tests with two years of data (pre-eradication vs one month, one year, three years, and five years post-eradication; one month vs one year post-eradication, etc.). We used a Bonferroni corrected alpha level of 0.005 to test for statistical significance and to account for multiple comparisons.

For the locally rare tree seedling plots with pre-eradication seedling data, we checked for over-dispersion and used a zero-inflated negative binomial generalized regression (to account for numerous zero counts within the data) [57] with Tukey HSD pairwise comparisons to examine datasets across sampling years.

To examine differences in native and non-native tree recruitment patterns across forest types (native, mixed, and non-native) in 2016 (five years post-eradication), we compared the number of *P*. *grandis* seedlings found on seedling transects in native (n = 17) and mixed (n = 14) forest types to the number found in non-native (n = 24) forests using a resampling approach. We sampled 10,000 means from the native and mixed forest data with replacement, and compared the means to zero (there were no seedlings found in non-native forests). In addition, we examined *C*. *nucifera* seedling recruitment across forest types (sample sizes are the same as above) using a zero-inflated negative binomial generalized regression with Tukey HSD pairwise comparisons.

We conducted all analyses using JMP Pro 13 statistical software (SAS Institute Inc. (2016), http://www.jmp.com), and we used an alpha level of 0.05 for all analyses, except where stated above.

## Results

### Native trees

**Fig 3:**
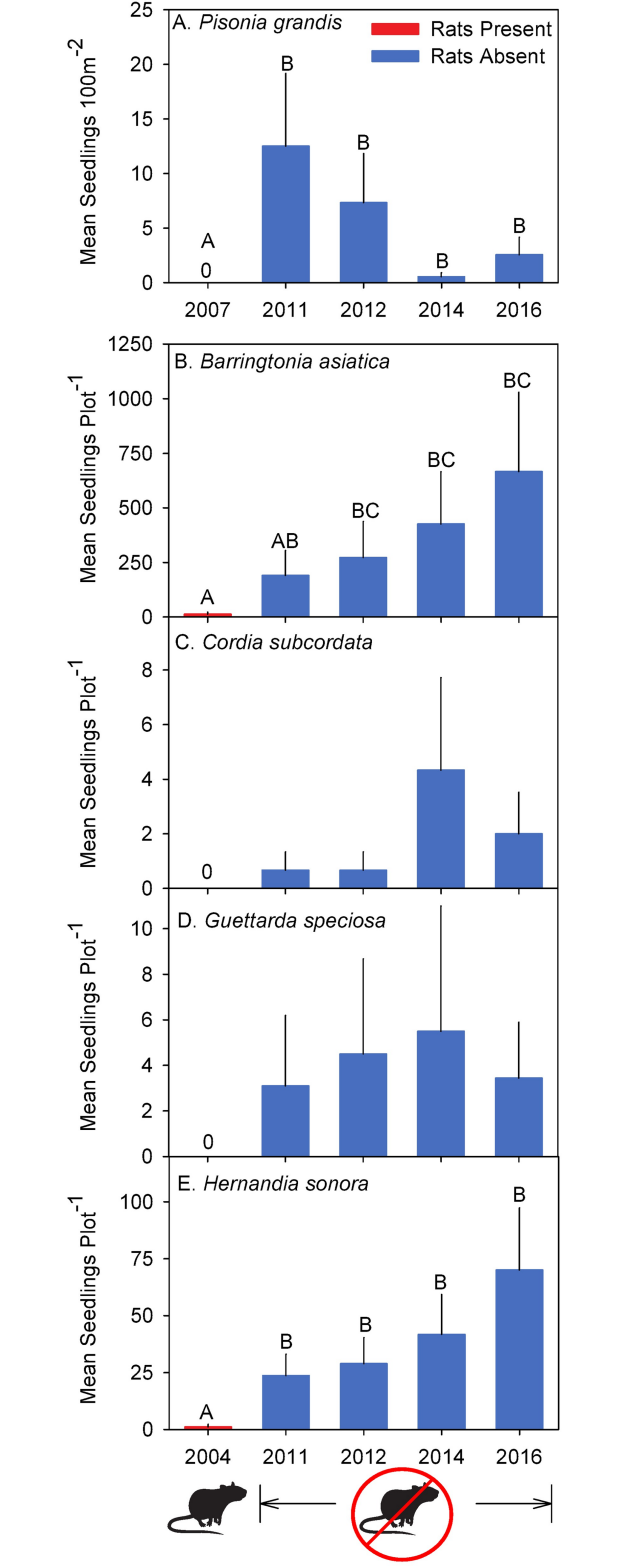
Changes in native species seedlings counts pre- and post- rat eradication. (A) Mean ± SE *Pisonia grandis* seedlings m^-2^ counted on 55 Seedling Transects across Palmyra Atoll pre- (2007) and post- (2011, 2012, 2014, and 2016) rat eradication. (B–E) Seedlings (Mean ± SE) counted per native locally rare tree seedling plot pre- (2004) and post- (2011, 2012, 2014, and 2016) rat eradication. *A—C* indicate significantly different data within each species grouping (after post-hoc correction; α = 0.05).

Using resampling, post-eradication seedling abundance of *P*. *grandis* was significantly greater across all years compared to pre-eradication (2011: *P* < 0.0001; 2012: *P* = 0.0001; 2014: *P* = 0.0134; 2016: *P* < 0.0001; Figs 1C and 3A). Pre-eradication, we found no *P*. *grandis* seedlings in all 55 seedling transects conducted across the three forest types. In contrast, we found a notable *P*. *grandis* germination event post- rat eradication with seedling counts averaging 12.5 seedlings transect^-1^ across all forest types one month following eradication (June 2011) (Fig 3A). While *P*. *grandis* seedling counts in 2014 and 2016 were reduced in comparison to the 2011 post-eradication germination event, we continued to observe recruitment (in comparison to no recruitment in pre-eradication surveys).

For two of the five rare native tree species, the mean number of seedlings observed within plots significantly increased during our five-year post-eradication monitoring period compared to pre-eradication levels (Fig 3B–3E). *Barringtonia asiatica* seedling abundance significantly increased one year (diff = -3.04, df = 46, t = -2.84, *P* = 0.05), three years (diff = -3.49, df = 46, t = -3.26, *P* = 0.0172) and five years (diff = -3.94, df = 46, t = -3.67, P = 0.0054) post-eradication (Fig 3B), while *H*. *sonora* seedling recruitment was significantly greater than pre-eradication levels every year post-eradication (2011: diff = -3.05, df = 66, t = -3.33, *P* = 0.012; 2012: diff = -3.22, df = 66, t = -3.51, *P* = 0.007; 2014: diff = -3.58, df = 66, t = -3.86, *P* = 0.0024; 2016: diff = -4.09, df = 66, t = -4.38, *P* = 0.0004; Fig 3E). For two additional rare native tree species where no seedlings were observed in pre-eradication monitoring (*C*. *subcordata* and *G*. *speciosa*), we observed seedlings during all post-eradication monitoring periods (Fig 3C and 3D), nevertheless, no statistical difference was evident in seedling counts pre- and post-eradication. For one native tree species, *N*. *oppositifolium*, we found no seedling recruitment within plots either pre- or post-eradication (not graphed).

### Non-native trees

**Fig 4:**
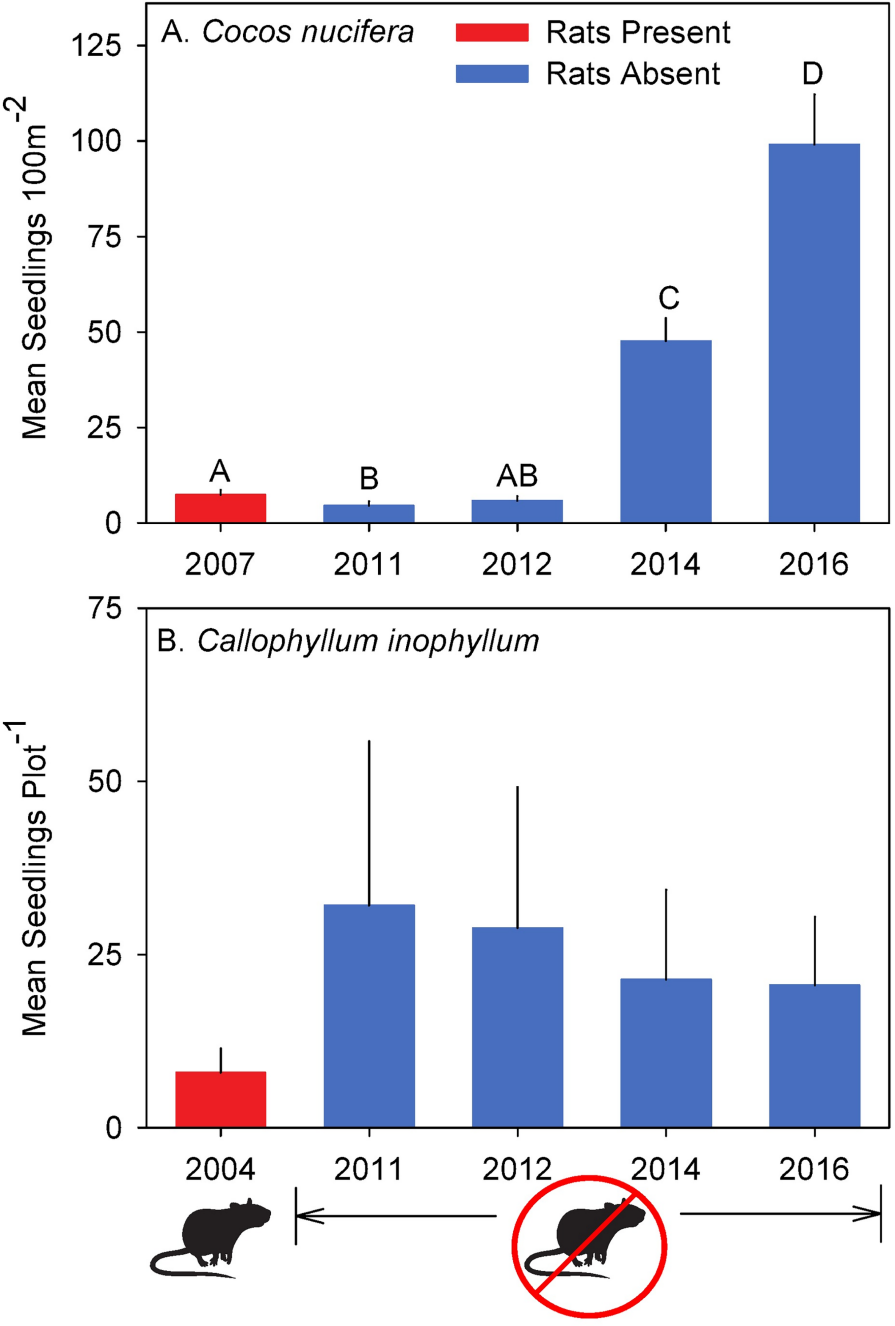
Changes in non-native species seedlings counts pre- and post- rat eradication. (A) Mean ± SE *Cocos nucifera* seedlings m^-2^ counted on 55 Seedling Transects across Palmyra Atoll pre- (2007) and post- (2011, 2012, 2014, and 2016) rat eradication. *A–D* indicate significantly different data (after multiple comparisons; α = 0.005). (B) Seedlings (Mean ± SE) counted per non-native (*Calophyllum inophyllum*) locally rare tree seedling plot pre- (2004) and post- (2011, 2012, 2014, and 2016) rat eradication were not significantly different (after post-hoc correction; α = 0.05).

*Cocos nucifera* seedling recruitment was overall significantly different pre vs. post rat eradication (F_(Greenhouse-Geisser)_ = 49.5097, df = 1.89, 102.31, *P*<0.0001). More specifically, *C*. *nucifera* seedling recruitment remained relatively low both pre-eradication and through 2012 (Fig 4A; Table 1). By 2014, seedling counts significantly increased (F_(Greenhouse-Geisser)_ = 33.8035, df = 1, 54, *P*<0.0001) and increased again in 2016 (F_(Greenhouse-Geisser)_ = 41.425, df = 1, 54, *P*<0.0001) when compared to pre-eradication counts (Fig 1D). In addition, seedling counts significantly increased in 2016 compared to 2014 (F_(Greenhouse-Geisser)_ = 9.9643, df = 1,54, *P*<0.0001; see Table 1 for all comparisons). By 2016, seedling counts were 13 times greater than those measured pre-eradication (Fig 4A).

**Table 1:** *Cocos nucifera* seedling transect repeated measures multiple comparison results.

Year	*F*_*(Greenhouse-Geisser)*_	Num. df	Den. df	*P > F*
2007 vs. 2011	9.1462	1	54	0.0038
2007 vs. 2012	2.7476	1	54	0.1032
2007 vs. 2014	33.8035	1	54	<0.0001
2007 vs. 2016	41.4250	1	54	<0.0001
2011 vs. 2012	6.4786	1	54	0.0138
2011 vs. 2014	70.8347	1	54	<0.0001
2011 vs. 2016	74.3930	1	54	<0.0001
2012 vs. 2014	65.7695	1	54	<0.0001
2012 vs. 2016	69.0316	1	54	<0.0001
2014 vs. 2016	9.9643	1	54	0.0026

Pre-eradication *C*. *nucifera* seedling counts were conducted in 2007, and post-eradication seedling counts were conducted in 2011, 2012, 2014, and 2016. Num. df and Den. df refer to numerator and denominator degrees of freedom, respectively. A Bonferroni corrected alpha level of 0.005 was used to test for statistical significance.

Seedling abundance for the rare non-native species, *C*. *inophyllum*, was not significantly different from pre-eradication levels any year post-eradication (Fig 4B).

### Comparative analysis

**Fig 5:**
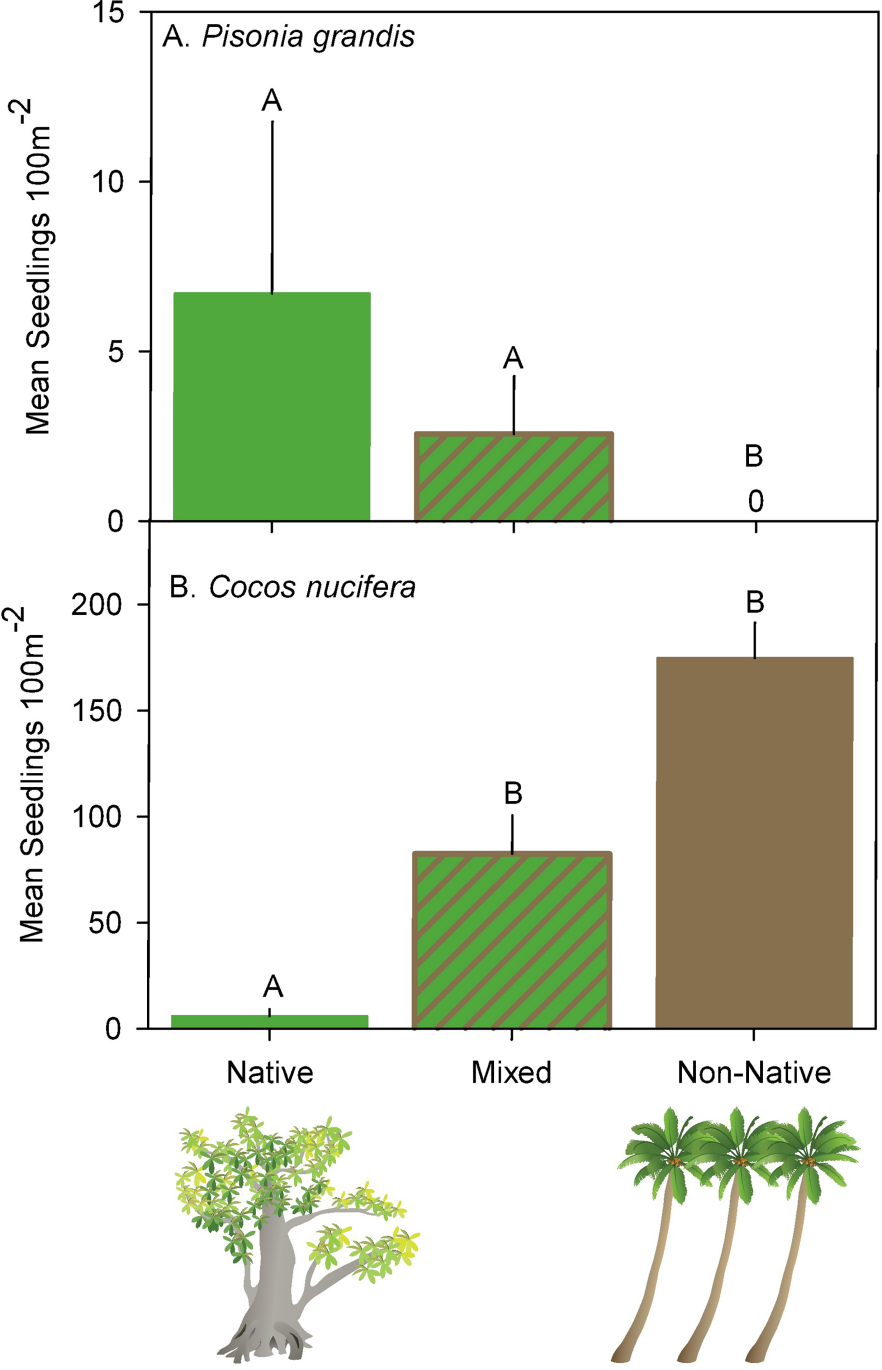
Common native and non-native plant species seedling counts within three forest types. (A) Mean ± SE *Pisonia grandis* seedlings m^-2^ counted on 55 Seedling Transects distributed across the three forest types (Native, Mixed, Non-Native) found on Palmyra Atoll five years post-eradication (2016). (B) Mean ± SE *Cocos nucifera* seedlings m^-2^ counted on 55 Seedling Transects distributed across the three forest types (Native, Mixed, Non-Native) found on Palmyra Atoll five years post-eradication (2016). *A*, *B* indicate significantly different data within each species grouping (after post-hoc correction; α = 0.05).

Examination of *P*. *grandis* and *C*. *nucifera* seedling counts within the three forest types five years post-eradication (2016) revealed important differences in native vs. non-native plant recruitment patterns (Fig 5). Native *P*. *grandis* recruitment was significantly different across forest types with relatively high levels of recruitment in both native dominated forests (using resampling, *P* = 0.0002) and mixed forests (*P* = 0.0102) when compared to non-native forests (where we saw no recruitment) (Fig 5A). In contrast, non-native *C*. *nucifera* recruitment was also significantly different across forest types but recruitment in non-native dominated forests was two and 29 times greater than that observed in mixed and native forests, respectively (mixed vs. native: diff = 2.02, df = 52, t = 5.03, *P* = < 0.0001; non-native vs. native: diff = 2.65, df = 52, t = 7.2, *P* = < 0.0001, Fig 5B).

## Discussion

Understanding the effects of rat eradication on plant communities is critical to understanding the long-term impacts of this increasingly common conservation intervention [2]. These responses are likely to be complex; short- and long-term responses of plants to rat eradication will be a function of the direct impact of rat herbivory and seed predation, the life history and reproductive strategy of individual plant species, environmental conditions, and interactive effects of other flora and fauna responding to the rat eradication [27]. Despite this complexity, we found that rat eradication was followed by increased recruitment for >80% of studied native tree species five years out, starting as early as one-month post-eradication. We also found increased recruitment of one important non-native palm, *C*. *nucifera*.

The increases in seedling recruitment we observed are consistent with previous studies identifying seed and seedling consumption by rats as a factor inhibiting plant recruitment on the atoll [33, 39] and elsewhere [8, 10, 12, 31]. Notably, while no seedlings of the highly abundant *P*. *grandis* were observed during pre-eradication monitoring, recruitment increased dramatically and immediately (one month) post- rat eradication, although this response diminished in later years. The strong increase in recruitment was likely due to the direct effects of rat predation on *P*. *grandis* seeds, which are a known food item for rats [35]. Prior to the eradication, *P*. *grandis* was recruiting primarily via vegetative propagation, with seed and seedling predation preventing seedling recruitment. By facilitating the establishment of *P*. *grandis* seedlings produced via sexual reproduction, the rat eradication may bolster the long-term survival of this plant in the face of ecosystem change [58].

There are multiple potential explanations for subsequent decline in seedling recruitment in *P*. *grandis* between one and five years post-eradication including: 1) competition with *C*. *nucifera* seedlings in the monodominant stands of coconut palms, particularly in the non-native and mixed forests, 2) intraspecific competition with established seedlings, 3) a period of high seed production (or ‘pulse’) coinciding with the eradication in 2011, and 4) post-eradication increases in the presence of omnivorous land crab species. Preliminary evidence exists for each explanation:

1. In forest habitats with the highest *C*. *nucifera* seedling establishment, *P*. *grandis* seedling recruitment is lowest (Fig 5A and 5B).
2. Although we did not follow individual *P*. *grandis* seedlings over time, incidental observations suggest that a subset of seedlings survived into following years, potentially shading out new *P*. *grandis* recruits (pers. obs.).
3. Both the timing and scale of *P*. *grandis* seed production is at least partially driven by precipitation and can vary wildly within and between years [56]. In this case, while *P*. *grandis* seedling establishment does appear to be influenced by the absence of rats, the high rates of production of seeds during our 2011 monitoring period was almost certainly unrelated to the rat eradication (as seeds would have been on trees prior to the eradication event). This strong pulse of seed production was unique among our post-eradication survey years.
4. Land crabs are significant seed predators and herbivores that are known to affect plant community structure on remote oceanic islands [59, 60]. On Palmyra Atoll, rats were known to limit crab numbers [33], and five years post-eradication the relative abundance of several crab species appears to be increasing [61], potentially leading to increased consumption of seeds and seedlings by land crabs. The impacts of crabs on seedling recruitment may continue to increase as their populations grow following release from invasive rat predation.

It is reasonable to assume that several of these mechanisms may influence seedling recruitment following rat eradications, and these remain important avenues for investigation.

The post-eradication increases in seedling recruitment observed in other native tree species (*B*. *asiatica*, *H*. *sonora*, *G*. *speciosa* and *C*. *subcordata*) were also likely the result of reduced seed or seedling predation from rats. Several of these species have been observed to be heavily predated by rats in this site or elsewhere in Polynesia [33, 34]. However, unlike *P*. *grandis*, *B*. *asiatica* and *H*. *sonora* were experiencing successful, if limited, recruitment prior to the rat eradication. One reason that these species were able to sustain recruitment in the presence of rats may be because the fruit structure of these species provided them greater relative protection from seed predation. While the *P*. *grandis* seed is surrounded only by a thin exocarp, *B*. *asiatica* contains the toxin saponin and *H*. *sonora* fruit has a thick hard exocarp. Still, given the very limited recruitment observed for these native species prior to rat eradication, we anticipate that rat eradication may allow long-term increases in the abundance of these native tree species.

Not all native tree species monitored during our study showed an increase in recruitment. We found no difference in seedling counts of *N*. *oppositifolium*, pre- vs. post-eradication, despite rats being a known seed predator of this species on other tropical islands [34]. Likewise, we found no change in seedling counts of the non-native tree *C*. *inophyllum*; this is unsurprising given that the fruit is known to be poisonous to rats [62]. The lack of change in recruitment of *N*. *oppositifolium* and *C*. *inophyllum* is consistent with observations that seed handling by rats and crabs is often non-lethal for these tree species [16, 33, 34].

The differential responses to rat eradication across plant species observed in this study are similar to results reported from rat eradications in very different contexts. For example, *R*. *exulans* and *R*. *norvegicus* were found to depress recruitment in only 11 of 17 species of coastal trees on 14 New Zealand offshore islands studied [13] and 3 of 22 woody or tree fern species on Ulva Island [28]. In order to move towards a more predictive capacity for evaluating the likely impacts of rat eradication efforts on plant communities, further research is needed to understand specific seed or seedling traits that drive differential responses among plants to rat removal.

As noted, tree seedling recruitment is complex and likely a response to a host of variables. Most notably, while Palmyra has a relatively constant warm, wet tropical climate, precipitation can vary widely between months and across years (S1 Fig) and could feasibly influence recruitment. However, there were no clear correlations between monthly precipitation and recruitment success. Moreover, given the extremely high rates of rainfall in the system (~4.5 m per year), it seems unlikely that recruitment of most plants is rainfall limited.

While the increased recruitment of native trees post- rat eradication suggests clear conservation benefits of rat eradication, we also found increased and accelerating recruitment of *C*. *nucifera* five years post-eradication, especially in non-native and mixed forests where adult *C*. *nucifera* were present. As with native species, this increase is likely due to release from seed predation by rats. *Cocos nucifera’s* particularly strong response is likely due to the fact that the seeds appear less vulnerable to predation by land crabs than are the smaller seeded native species (such that there is little compensatory predation of seeds by crabs in the absence of rats). Unlike for native species, the strong response of *C*. *nucifera* took several years to emerge. This is likely because *C*. *nucifera’s* slow seed production requires approximately a year for the seed to reach maturity [63] and most of rat predation occurred on juvenile seeds; consequently, the longer monitoring period (i.e. five years) was more relevant for detecting the significant increase in seedling recruitment post-eradication.

High abundances of *C*. *nucifera* have been shown to cause cascading ecosystem impacts including changes in water availability, soil nutrient content, plant and animal community composition, and changes in the behavior, size, and body condition of island fauna [45]. If uncontrolled, non-native *C*. *nucifera* forests will likely expand on Palmyra Atoll with related detrimental impacts to native plant and animal communities and ecosystem processes that may limit the larger beneficial impacts of rat eradication [64]. Multiple direct and indirect factors will determine long-term changes in native plant community structure including recovery of native crab populations, inter- and intraspecific competition among the plant species, changes in insect community structure, and seabird-mediated nutrient availability. Understanding responses to invasive animal eradication requires a whole-ecosystem context, including responses of both native and non-native species [65] to evaluate effectiveness of rat eradication and gauge other management actions required to promote the long-term recovery of native plant species. Our study contributes towards this knowledge base by presenting evidence of how tree and palm seedlings respond to the release from rat predation on a tropical atoll.

## Conclusions

Our study adds to the growing literature on the potential benefits of rat eradication; in this case, we document strong apparent release of native trees following removal of rat herbivory and seed predation. To the best of our knowledge, this is the first study to investigate seedling recruitment following a rat eradication on a tropical island. Given the importance of native trees for both vertebrate and invertebrates, this may foreshadow strong positive impacts of the eradication for many other native species at Palmyra Atoll. However, consistent with management expectations during the eradication planning, we also detected a strong increase in *C*. *nucifera* seedlings, highlighting potential negative impacts via competition with native tree species and loss of habitat. As a result, active management of *C*. *nucifera* will likely be needed to control proliferation of this species, and maximize benefits to native vegetation for the atoll [51].

The potential long-term effects of this rat eradication on plant communities are just beginning to unfold. We only examined short-term effects on seedling establishment, which, while critical to long-term persistence of various tree species, may only reflect transient dynamics. Rats also, no doubt, directly affected vertebrate and invertebrate communities through predation and competition [3, 66], including crabs and seabirds. As these populations respond to the rat eradication, indirect effects may become more important in determining the future of tree regeneration at Palmyra and ultimately mature forest composition. Monitoring must continue to play a key role in documenting long-term effects of this eradication and to facilitate early intervention if any negative outcomes emerge. Cumulatively, these results, provide much needed insight into how tropical island ecosystems will likely respond to conservation actions like rodent eradications [67] or to future rat invasions into insular systems.

## Supporting information

S1 Table Count of locally rare tree seedling plots. (DOCX)

S1 Fig Fifteen years of precipitation on Palmyra Atoll. Rainfall on Palmyra Atoll from 2002 to 2017. Survey month and two months prior to the survey period are highlighted (red dots = pre-eradication and blue dots = post-eradication). Horizontal lines indicate average rainfall and one standard deviation.
(TIF)

## References

[1] Atkinson IAE. The spread of commensal species of Rattus to oceanic islands and their effects on island avifaunas In: Moors PJ, editor. Conservation of Island Birds. New Zealand: Department of Scientific and Industrial Research; 1985 p. 35–81.

[2] Towns DR, Atkinson IAE, Daugherty CH. Have the harmful effects of introduced rats on islands been exaggerated? Biol Invasions. 2006;8(4): 863–91.

[3] Jones HP, Tershy BR, Zavaleta ES, Croll DA, Keitt BS, Finkelstein ME, et al Severity of the effects of invasive rats on seabirds: A global review. Conserv Biol. 2008;22(1): 16–26. 10.1111/j.1523-1739.2007.00859.x 18254849

[4] Doherty TS, Glen AS, Nimmo DG, Ritchie EG, Dickman CR. Invasive predators and global biodiversity loss. Proceedings of the National Academy of Sciences of the United States of America. 2016;113(40): 11261–5. 10.1073/pnas.1602480113 27638204 PMC5056110

[5] Croll DA, Maron JL, Estes JA, Danner EM, Byrd GV. Introduced predators transform subarctic islands from grassland to tundra. Science. 2005;307(5717): 1959–61. 10.1126/science.1108485 15790855

[6] Mulder CPH, Grant-Hoffman MN, Towns DR, Bellingham PJ, Wardle DA, Durrett MS, et al Direct and indirect effects of rats: does rat eradication restore ecosystem functioning of New Zealand seabird islands? Biol Invasions. 2009;11(7): 1671–88.

[7] Le Corre M, Danckwerts DK, Ringler D, Bastien M, Orlowski S, Morey Rubio C, et al Seabird recovery and vegetation dynamics after Norway Rat eradication at Tromelin Island, western Indian Ocean. Biol Conserv. 2015;185: 85–94.

[8] Grant-Hoffman MN, Mulder CP, Bellingham PJ. Invasive rats alter woody seedling composition on seabird-dominated islands in New Zealand. Oecologia. 2010;163(2): 449–60. 10.1007/s00442-009-1523-6 20033216

[9] Shiels AB, Pitt WC, Sugihara RT, Witmer GW. Biology and impacts of Pacific Island invasive species. 11. *Rattus rattus*, the Black Rat (Rodentia: Muridae). Pac Sci. 2014;68(2): 145–84.

[10] Traveset A, Nogales M, Alcover JA, Delgado JD, López-Darias M, Godoy D, et al A review on the effects of alien rodents in the Balearic (Western Mediterranean Sea) and Canary Islands (Eastern Atlantic Ocean). Biol Invasions. 2009;11(7): 1653–70.

[11] Pender R, Shiels A, Bialic-Murphy L, Mosher S. Large-scale rodent control reduces pre- and post-dispersal seed predation of the endangered Hawaiian lobeliad, Cyanea superba subsp. superba (Campanulaceae). Biol Invasions. 2013;15(1): 213–23.

[12] Auld T, Hutton I, Ooi MJ, Denham A. Disruption of recruitment in two endemic palms on Lord Howe Island by invasive rats. Biol Invasions. 2010;12(9): 3351–61.

[13] Campbell DJ, Atkinson IAE. Depression of tree recruitment by the Pacific rat (*Rattus exulans* Peale) on New Zealand's northern offshore islands. Biol Conserv. 2002;107(1): 19–35.

[14] Hunt TL. Rethinking Easter Island's ecological catastrophe. Journal of Archaeological Science. 2007;34(3): 485–502.

[15] Grant-Hoffman M, Mulder CH, Bellingham P. Effects of invasive rats and burrowing seabirds on seeds and seedlings on New Zealand islands. Oecologia. 2010;162(4): 1005–16. 10.1007/s00442-009-1500-0 19921273

[16] Meyer J-Y, Butaud J-F. The impacts of rats on the endangered native flora of French Polynesia (Pacific Islands): drivers of plant extinction or *coup de grâce* species? Biol Invasions. 2009;11: 1569–85.

[17] Newton KM, McKown M, Wolf C, Gellerman H, Coonan T, Richards D, et al Response of Native Species 10 Years After Rat Eradication on Anacapa Island, California. Journal of Fish and Wildlife Management. 2016;7(1): 72–85.

[18] de Wit LA, Croll DA, Tershy B, Newton KM, Spatz DR, Holmes ND, et al Estimating Burdens of Neglected Tropical Zoonotic Diseases on Islands with Introduced Mammals. The American journal of tropical medicine and hygiene. 2017;96(3): 749–57. 10.4269/ajtmh.16-0573 28138052 PMC5361556

[19] Russell JC, Holmes ND. Tropical island conservation: rat eradication for species recovery. Biol Conserv. 2015;185: 1–7.

[20] St Clair JJH, Poncet S, Sheehan DK, Szekely T, Hilton GM. Responses of an island endemic invertebrate to rodent invasion and eradication. Anim Conserv. 2011;14(1): 66–73.

[21] Monks JM, Monks A, Towns DR. Correlated recovery of five lizard populations following eradication of invasive mammals. Biol Invasions. 2014;16(1): 167–75.

[22] Bellingham PJ, Towns DR, Cameron EK, Davis JJ, Wardle DA, Wilmhurst JM, et al New Zealand island restoration: seabirds, predators, and the importance of history. N Z J Ecol. 2010;34: 115–36.

[23] Lorvelec O, Pascal M. French attempts to eradicate non-indigenous mammals and their consequences for native biota. Biol Invasions. 2005;7: 135–40.

[24] Whitworth DL, Carter HR, Gress F. Recovery of a threatened seabird after eradication of an introduced predator: Eight years of progress for Scripps's murrelet at Anacapa Island, California. Biol Conserv. 2013;162: 52–9.

[25] Brooke MdL, Bonnaud E, Dilley BJ, Flint EN, Holmes ND, Jones HP, et al Seabird population changes following mammal eradications on islands. Anim Conserv. 2017; 10.1111/acv.12344

[26] Jones HP, Holmes ND, Butchart SHM, Tershy BR, Kappes PJ, Corkery I, et al Invasive mammal eradication on islands results in substantial conservation gains. Proceedings of the National Academy of Sciences of the United States of America. 2016;113: 4033–8. 10.1073/pnas.1521179113 27001852 PMC4839448

[27] Schweizer D, Jones HP, Holmes ND. Literature Review and Meta-Analysis of Vegetation Responses to Goat and European Rabbit Eradications on Islands. Pac Sci. 2015;70(1): 55–71.

[28] Clayton RI, Wilson DJ, Dickinson KJM, West CJ. Response of seedling communities to mammalian pest eradication on Ulva Island, Rakiura National Park, New Zealand. N Z J Ecol. 2008;32(1): 103–7.

[29] Allen RB, Lee WG, Rance BD. Regeneration in indigenous forest after eradication of Norway Rats, Breaksea Island, New Zealand. N Z J Bot. 1994;32(4): 429–39.

[30] Campbell DJ. Changes in numbers of woody plant seedlings on Kapiti Island after rat eradication Wellington, NZ: Department of Conservation; 2002.

[31] Grant-Hoffman MN, Barboza P. Herbivory in invasive rats: criteria for food selection. Biol Invasions. 2010;12(4): 805–25.

[32] Fall MW, Medina AB, Jackson WB. Feeding Patterns of Rattus rattus and Rattus exulans on Eniwetok Atoll, Marshall Islands. J Mammal. 1971;52(1): 69–76. 5545566

[33] Wegmann AS. Limitations to tree seedling recruitment at Palmyra Atoll Honolulu, Hawai'i: University of Hawai'i; 2009.

[34] McConkey KR, Drake DR, Meehan HJ, Parsons N. Husking stations provide evidence of seed predation by introduced rodents in Tongan rain forests. Biol Conserv. 2003;109(2): 221–5.

[35] Caut S, Angulo E, Courchamp F. Dietary shift of an invasive predator: rats, seabirds and sea turtles. J Appl Ecol. 2008;45(2): 428–37. 10.1111/j.1365-2664.2007.01438.x 18784794 PMC2326384

[36] Allaway WG, Ashford AE. Nutrient input by seabirds to the forest on a coral island of the Great Barrier Reef. Mar Ecol Prog Ser. 1984;19: 297–8.

[37] Briggs AA, Young HS, McCauley DJ, Hathaway SA, Dirzo R, Fisher RN. Effects of spatial subsidies and habitat structure on abundance and ecology of two congeneric gecko species. Plos One. 2012;7(8): e41364 10.1371/journal.pone.0041364 22899995 PMC3416811

[38] Young HS, McCauley DJ, Dunbar RB, Hutson MS, Miller Ter-Kuile A, Dirzo R. The roles of productivity and ecosystem size in determining food chain length in tropical terrestrial ecosystems. Ecology. 2013;94(3): 692–701. 23687895 10.1890/12-0729.1

[39] Young HS, McCauley DJ, Guevara R, Dirzo R. Consumer preference for seeds and seedlings of rare species impacts tree diversity at multiple scales. Oecologia. 2013;172(3): 857–67. 10.1007/s00442-012-2542-2 23229391

[40] Walker TA. Part I. The distribution, abundance and dispersal by seabirds of *Pisonia grandis* In Pisonia islands of the Great Barrier Reef. Atoll Res Bull. 1991;350.

[41] Mueller-Dombois D, Fosberg FR. Vegetation of the tropical Pacific islands New York: Springer; 1998.

[42] Handler AT, Gruner DS, Haines WP, Lange MW, Kaneshiro KY. Arthropod surveys on Palmyra Atoll, Line Islands, and insights into the decline of the native tree *Pisonia grandis* (Nyctaginaceae). Pac Sci. 2007;61(4): 485–502.

[43] Hower LM, Hedges SB. Molecular phylogeny and biogeography of West Indian Teiid lizards of the genus Ameiva. Caribb J Sci. 2003;39(3): 298–306.

[44] Gunn BF, Baudouin L, Olsen KM. Independent Origins of Cultivated Coconut (Cocos nucifera L.) in the Old World Tropics. PLOS ONE. 2011;6(6): e21143 10.1371/journal.pone.0021143 21731660 PMC3120816

[45] Young HS, Miller-ter Kuile A, McCauley DJ, Dirzo R. Cascading community and ecosystem consequences of introduced coconut palms (*Cocos nucifera*) in tropical islands. Canadian Journal of Zoology. 2016;95(3): 139–48.

[46] Krauss KW, Duberstein JA, Cormier N, Young HS, Hathaway SA. Proximity to encroaching coconut palm limits native forest water use and persistence on a Pacific atoll. Ecohydrology. 2015;8(8): 1514–24.

[47] Matisoo-Smith E, Robins JH. Origins and dispersals of Pacific peoples: Evidence from mtDNA phylogenies of the Pacific rat. Proceedings of the National Academy of Sciences of the United States of America. 2004;101(24): 9167–72. 10.1073/pnas.0403120101 15184658 PMC428491

[48] Depkin CD. Trip report to Palmyra Atoll NWR, 06 August 2001 to 07 October 2002 Honolulu, Hawaii: U.S. Fish and Wildlife Service, 2002.

[49] Jackson WB. Productivity in high and low islands with special emphasis to rodent populations. Micronesica. 1967;3(15): 5–15.

[50] Wegmann AS, Flint E, White S, Fox M, Howald G, McClelland P, et al. Pushing the envelope in paradise: a novel approach to rat eradication at Palmyra Atoll. Vertebrate Pest Conference; Monterey, California, USA: University of California, Davis; 2012. p. 48–53.

[51] USFWS. Palmyra Atoll National Wildlife Refuge Rat Eradication Project Final Environmental Impact Statement Honolulu, Hawaii: US Fish & Wildlife Service, 2011.

[52] Young HS, Raab TK, McCauley DJ, Briggs AA, Dirzo R. The coconut palm, *Cocos nucifera*, impacts forest composition and soil characteristics at Palmyra Atoll, Central Pacific. Journal of Vegetation Science. 2010;21(6): 1058–68.

[53] Pain D, de L. Brooke M, K. Finnie J, Jackson A. Effects of Brodifacoum on the Land Crab of Ascension Island2000. 380 p.

[54] Pitt WC, Berentsen AR, Shiels AB, Volker SF, Eisemann JD, Wegmann AS, et al Non-target species mortality and the measurement of brodifacoum rodenticide residues after a rat (Rattus rattus) eradication on Palmyra Atoll, tropical Pacific. Biol Conserv. 2015;185: 36–46.

[55] US EPA. United States Environmental Protection Agency, Reregistration Eligibility Decision (RED) Rodenticide Cluster. Prevention, Pesticides and Toxic Substances (7508W). EPA 738-R-98-007: 1998.

[56] Burger AE. Dispersal and germination of seeds of *Pisonia grandis*, an Indo-Pacific tropical tree associated with insular seabird colonies. J Trop Ecol. 2005;21: 263–71.

[57] Yau KKW, Wang K, Lee AH. Zero-Inflated Negative Binomial Mixed Regression Modeling of Over-Dispersed Count Data with Extra Zeros. Biometrical Journal. 2003;45(4): 437–52.

[58] Lei SA. Benefits and Costs of Vegetative and Sexual Reproduction in Perennial Plants: A Review of Literature. Journal of the Arizona-Nevada Academy of Science. 2010;42(1): 9–14.

[59] Green PT, O’Dowd DJ, Lake PS. Control of seedling recruitment by land crabs in rain forest on a remote oceanic island. Ecology. 1997;78(8): 2474–86.

[60] Lindquist ES, Krauss KW, Green PT, O'Dowd DJ, Sherman PM, Smith TJ. Land crabs as key drivers in tropical coastal forest recruitment. Biological Reviews. 2009;84(2): 203–23. 19391202 10.1111/j.1469-185x.2008.00070.x

[61] Nigro KM, Hathaway SA, Wegmann AS, Miller-ter Kuile A, Fisher RN, Young HS. Stable isotope analysis as an early monitoring tool for community-scale effects of rat eradication. Restor Ecol. 2017; 10.1111/rec.12511

[62] Burkill HM. The useful plants of West Tropical Africa 2nd edition ed. Oxon, UK: Royal Botanic Gardens Kew, CAB International; 1994.

[63] Chan E, Elevitch CR. *Cocos nucifera* (coconut) In: Elevitch CR, editor. Species Profiles for Pacific Island Agroforestry (http://www.traditionaltree.org), http://www.traditionaltree.org. 2.1 ed. Permanent Agriculture Resources (PAR), Holualoa, Hawaii: Traditional Tree Initiative; 2006 p. 1–27.

[64] McCauley DJ, DeSalles PA, Young HS, Dunbar RB, Dirzo R, Mills MM, et al From wing to wing: the persistence of long ecological interaction chains in less-disturbed ecosystems. 2012;2: 409. 10.1038/srep00409 PMC3354671 22624091

[65] Zavaleta ES, Hobbs RJ, Mooney HA. Viewing invasive species removal in a whole-ecosystem context. Trends Ecol Evol. 2001;16(8): 454–9.

[66] St Clair JJH. The impacts of invasive rodents on island invertebrates. Biol Conserv. 2011;144(1): 68–81.

[67] Caujapé-Castells J, Tye A, Crawford DJ, Santos-Guerra A, Sakai A, Beaver K, et al Conservation of oceanic island floras: Present and future global challenges. Perspectives in Plant Ecology, Evolution and Systematics. 2010;12(2): 107–29.

