# Supplementary material for: Invasive rat eradication strongly impacts plant recruitment on a tropical atoll
Source: PLoS One. 2018 Jul 17;13(7):e0200743. doi: 10.1371/journal.pone.0200743 (PMC6049951; doi:10.1371/journal.pone.0200743)
Supplement: S1 Table — (DOCX) [file pone.0200743.s001.docx]

| **Species** | **Pre-eradication** | **Post-eradication** | | | |
| --- | --- | --- | --- | --- | --- |
|  | **2004** | **2011** | **2012** | **2014** | **2016** |
| *Barringtonia asiatica* | 10 | 10 | 10 | 10 | 10 |
| *Hernandia sonora* | 9 | 16 | 16 | 15 | 15 |
| *Guettarda speciosa* | 11 | 10 | 10 | 10 | 9 |
| *Neisosperma oppositifolium* | 5 | 4 | 4 | 4 | 4 |
| *Cordia subcordata* | 3 | 3 | 3 | 3 | 3 |
| *Calophyllum inophyllum* | 11 | 12 | 12 | 12 | 12 |
| **Total Trees Sampled** | 49 | 55 | 55 | 54 | 53 |
