# Supplementary figures and images for: Invasive rat eradication strongly impacts plant recruitment on a tropical atoll
Source: PLoS One. 2018 Jul 17;13(7):e0200743. doi: 10.1371/journal.pone.0200743 (PMC6049951; doi:10.1371/journal.pone.0200743)

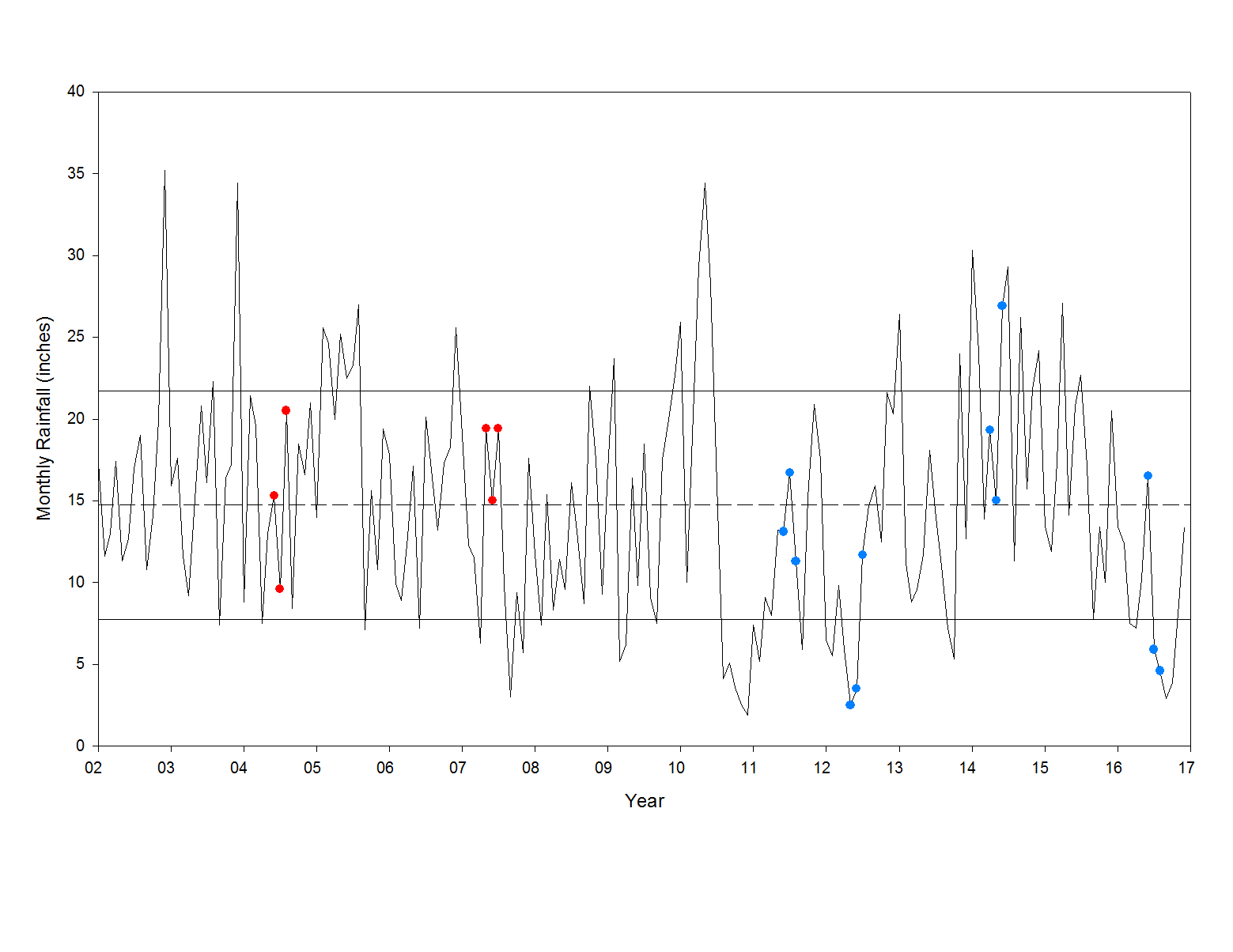

Supplement: S1 Fig — Rainfall on Palmyra Atoll from 2002 to 2017. Survey month and two months prior to the survey period are highlighted (red dots = pre-eradication and blue dots = post-eradication). Horizontal lines indicate average rainfall and one standard deviation. (TIF) [file pone.0200743.s002.TIF]
